# Marker-Assisted Backcross Breeding of Drought-Tolerant Maize Lines Transformed by Vacuolar H^+^-Pyrophosphatase Gene (*AnVP1*) from *Ammopiptanthus nanus*

**DOI:** 10.3390/plants14060926

**Published:** 2025-03-15

**Authors:** Yang Cao, Haoqiang Yu, Xin Guo, Yanli Lu, Wanchen Li, Fengling Fu

**Affiliations:** Key Laboratory of Biology and Genetic Improvement of Maize in Southwest Region, Ministry of Agriculture, Maize Research Institute, Sichuan Agricultural University, Chengdu 611130, China; caoy0112@163.com (Y.C.); gx1789862@163.com (X.G.); luyanli@sicau.edu.cn (Y.L.)

**Keywords:** agronomic trait, *Ammopiptanthus nanus*, drought stress, maize, molecular design breeding, vacuolar H^+^-pyrophosphatase

## Abstract

Maize is highly sensitive to water deficit but has high transpiration and biomass production, leading to a substantial water demand. Genetic engineering can overcome reproductive isolation and utilize drought-tolerant genes from distant species. *Ammopiptanthus nanus* is a relic of the Tertiary glaciation that can adapt to harsh environmental conditions. In our previous study, five maize homozygous T_8_ lines overexpressing the *AnVP1* gene from *Ammopiptanthus nanus* were generated and showed the enhancement of drought tolerance. However, the recipient inbred line Zh-1 was poor in yield and agronomic performance. In the present study, the *AnVP1* gene was backcrossed from donor parent L10 (one of the five T_8_ lines) into recurrent parent Chang 7-2 (one of the elite parents of the commercial hybrid Zhengdan 958). In total, 103 InDel markers were developed and used for assisted background selection. After two generations of foreground selection through glufosinate spraying, the detection of CP4 EPSP MAb1 strips, and the PCR amplification of the *AnVP1* gene, along with the similarity of agronomic traits to the recurrent parent, and background selection assisted by these InDel markers, the transgenic *AnVP1* gene became homozygous in the BC_2_ lines. The average recovery rate of the genetic background of the recurrent parent reached 74.80% in the BC_1_ population and 91.93% in the BC_2_ population, respectively. The results of RT-PCR and RT-qPCR indicated the stable expression of the *AnVP1* gene in the two ultimately selected BC_2_F_3_ lines, BC_2_-36-12 and BC_2_-5-15. The drought tolerance of these two BC_2_F_3_ lines were significantly improved compared to the recurrent parent Chang 7-2, as revealed by their wilting phenotype and survival rate of seedlings. This improvement was related to the enhancement of water-retention ability, as indicated by higher RWC and the reduction in damage, as shown by the decrease in REL, MDA, and H_2_O_2_ under drought stress. The result of field evaluation in two arid and semi-arid environments indicated that the drought tolerance of Chang 7-2 was significantly improved. This study suggests that the improved Chang 7-2 can be crossed with Zheng 58 to develop the transgenic commercial hybrid Zhengdan 958.

## 1. Introduction

Maize, originating from the humid and hot lowland tropics in South America, is well adapted to such environments but highly sensitive to water deficit [1,2]. Maize requires a substantial amount of water for growth due to its high transpiration rate and significant biological yield [3,4]. Although drought tolerance is a crucial trait in maize improvement, conventional breeding has made limited progress because of the constrains of germplasm resources [5,6,7,8,9]. Transgenic technology, however, can surmount reproductive isolation and make use of drought-tolerant genes from distantly related species [10,11,12,13]. According to the 2019 statistics of the International Service for the Acquisition of Agri-biotech Applications (ISAAA) (https://www.isaaa.org/, accessed on 14 August 2024), seven transgenic events that ectopically express bacterial genes such as cold shock protein (CSP) and choline dehydrogenase (CHDH), along with dozens of maize hybrids derived from them, have been approved for commercial release by the governments of various countries following drought tolerance evaluation and safety assessment [14,15]. Their application has significantly contributed to ensuring food security in the arid and semi-arid regions of Africa, Southeast Asia, and South America [16].

*Ammopiptanthus nanus*, a relic species from the Tertiary glaciation period, is mainly distributed at the junction of the Tarim Basin and the Pamir Plateau [17]. This tenacious species has evolved a remarkable tolerance to the harsh environment characterized by extreme drought and temperature variations [18]. Several of its genes, including those encoding the antifreeze protein (AFP), betaine aldehyde dehydrogenase (BADH), molybdenum cofactor sulfide (MCSU), expansin (EXPA), galactinol synthase (GS), and vacuolar H^+^-pyrophosphatase (VP), have been cloned and assessed for abiotic stress tolerance through ectopic expression in transgenic plants [19,20,21,22,23,24]. VP hydrolyzes pyrophosphatase (PPI) into two inorganic phosphate molecules and cooperates with vacuolar H^+^-adenosine triphosphatase (H^+^-ATPase) to transport excess H^+^ from the cytoplasm into the vacuole. This process establishes transmembrane H^+^ gradients and electrochemical potential, thereby maintaining ion homeostasis between the cytoplasm and the vacuole [25,26].

To date, embryonic calli induced from immature embryos remain the primary recipients for transgenic manipulation in maize. However, their induction and regeneration are highly genotype-dependent, primarily restricted to a few inbred lines such as A188, B73, and their hybrid Hi-II [27,28,29,30,31]. The agronomic traits of these inbred lines often fail to meet the requirements of commercial agricultural production [32,33,34,35,36]. Therefore, transgenes must be integrated into the genetic background of elite commercial inbred lines, which serve as recurrent parents. These recurrent parents are hybridized with recipient inbred lines, followed by successive backcrossing with their hybrid progeny to ensure genetic stability [34,36]. The presence of transgenes and their homozygosity in the backcross progenies are detected through resistance screening, PCR amplification, and phenotyping of the agronomic traits that closely resemble those of the recurrent parents. Marker-assisted selection (MAS) expedites the background recovery of recurrent parents (background selection) [36,37,38,39]. Available DNA markers include single-nucleotide polymorphisms (SNPs), simple sequence repeats (SSRs), insertion–deletion (InDel), and sequence-tagged microsatellite sites (STMSs) [36,37,38,40]. According to the investigation by Bregitzer et al. [41], a single round of marker-assisted backcrossing combined with selfing can achieve approximately a 75% background recovery rate. In practical applications, at least two rounds of backcrossing are typically required, using markers evenly distributed across the genome (one marker per 15–20 cM) [38,40]. In the biofortification project of ‘Golden Rice’, the recipient variety Kaybonnet exhibited poor yield and agronomic performance. Additionally, integration of the T-DNA into the promoter region of the endogenous *OsAux1* gene, which encodes an auxin transmembrane transporter, caused hormone imbalance and growth deformities in the transgenic event GR2-R1. Thus, the maize phytoene synthase (PSY) gene *ZmPsy* and the bacterium carotene desaturase (Crt) gene *CrtI* were transferred into the elite variety Swarna through backcrossing assisted by STMS markers. This approach resulted in the development of a vitamin A-fortified ‘Golden Rice’ variety with a background recovery rate of 96% and normal agronomic traits, successfully isolated from the BC_4_F_2_ population [34]. Similarly, in efforts to transfer the carotene desaturase gene *CrtRB1* into two recurrent inbred lines of maize, 106 and 214 SNP markers were used for assisted selection. At the BC_2_ generation, background recovery rates of the recurrent parents reached 78.83~99.44% and 87.26~92.42%, respectively, while β-carotene content was significantly enhanced through foreground selection [42,43].

InDel polymorphisms represent a major source of genomic evolutionary variation. Due to their multi-allelic and co-dominant nature, InDels provide more comprehensive genomic information compared to bi-allelic markers such SNPs, SSRs, and STMSs [44,45,46]. Additionally, InDels are relatively easy to detect, making them highly useful for genetic studies. InDel markers have been extensively applied in molecular evolution studies, population genetic analysis, genetic map construction, quantitative trait locus (QTL) mapping, and MAS [47,48,49,50]. Given their technological advantages and proven effectiveness in MAS for QTL background selection, InDel markers hold great potential as a powerful tool for the background selection of transgene backcrossing.

In our previous study, five homozygous T_8_ lines of maize were screened from 22 independent transgenic events, each carrying a single copy of the *AnVP1* gene from *Ammopiptanthus nanus*. The ectopic expression of *AnVP1* in these lines was confirmed through real-time quantitative PCR (RT-qPCR), transcriptome analysis, and an enzyme-linked immunosorbent assay (ELISA), followed by an evaluation of their enhanced drought tolerance [24]. However, the recipient inbred line Zh-1 exhibited poor yield and suboptimal agronomic performance. In the present study, the *AnVP1* gene was integrated from donor parent L10—the most drought-tolerant among the five T_8_ lines—into the recurrent parent Chang 7-2. As one of the elite parents of the widely cultivated commercial hybrid Zhengdan 958, Chang 7-2 possesses desirable agronomic traits but require improvement in drought tolerance [51]. Background selection was conducted using InDel markers designed based on genome sequence polymorphism between the donor (L10) and the recurrent parent (Chang 7-2). Two BC_2_F_3_ lines, BC_2_-36-12 and BC_2_-5-15, were screened. These lines showed the enhancement of drought tolerance, characterized by minimal wilting phenotypes, higher survival rate and relative water content (RWC), lower relative electrolyte leakage (REL), malondialdehyde (MDA), and hydrogen peroxide (H_2_O_2_), and higher yields under drought conditions. This study suggests that the improved Chang 7-2 can be hybridized with the other parent Zheng 58 to generate the transgenic commercial hybrid Zhengdan 958.

## 2. Results

### 2.1. Polymorphic InDel Markers

After quality filtering, alignment to the B73 reference genome sequences, and the removal of repeat sequences, a total of 73,029 length variation sites were identified between the genome sequences of inbred lines Chang 7-2 and Zh-1 using HaplotypeCaller software (v4.1.4.1). These sites were further filtered using VCFtools with a genotype quality (GO) threshold of >30. Among them, 1749 InDel sites with a length difference ranging from 30 to 50 bp and a G/C ratio between 55% and 60% were screened. From them, 103 InDel markers with intervals of approximately 20 cM were screened and roughly evenly distributed across the 10 chromosomes of the maize genome (Figure S1). Primers were designed for these markers and subsequently used to amplify the genomic DNA of Chang 7-2 and Zh-1 (Table S1). The results of agarose gel electrophoresis indicated that the amplified fragments were 100 to 200 bp in length and exhibited polymorphic InDel markers between Chang 7-2 and Zh-1 (Figure S2). These markers were evaluated to be suitable for the backcrossing of the *AnVP1* gene from the homozygous transgenic lines of Zh-1 to the recurrent parent Chang 7-2.

### 2.2. Homozygous BC_2_F_3_ Lines

**Figure 1:**
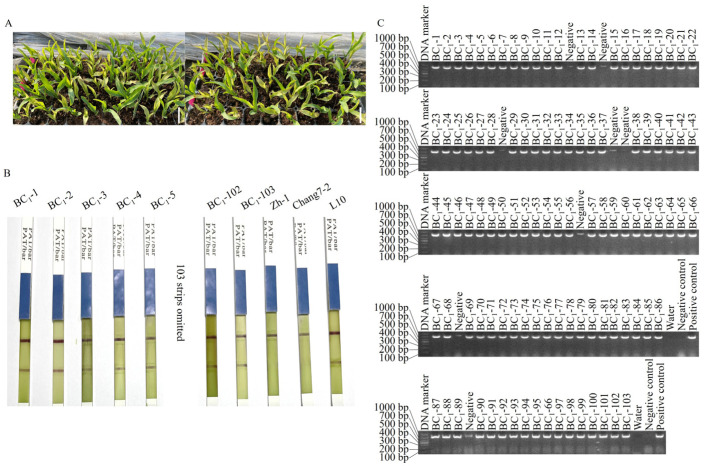
Segregation of wilting and normal growing seedlings of BC_1_ population (**A**), the positive detection of partial BC_1_ seedlings by CP4 EPSP MAb1 strips (**B**) and agarose electrophoresis of the transgene fragment amplified from the BC_1_ seedlings (**C**). The negative control is the DNA samples extracted from the negative seedlings identified by CP4 ESPSP Mab1 strips. The scale bar is 5 cm.

On the fifth day after glufosinate spraying, 111 seedlings in the BC_1_ population between Chang 7-2 and the homozygous T_8_ line L10 exhibited normal growth, whereas the remaining 113 seedlings began wilting (Figure 1A). The segregation ratio was 111:113 with a calculated χ^2^ = 0.01 (χ^2^ (0.05, 1) = 3.84), indicating its accordance with the theoretical ratio of 1:1. Among the normally growing seedlings, 103 positive seedlings were identified using CP4 EPSP MAb1 strips (Figure 1B) and by PCR amplification of the transgenic *AnVP1* gene (Figure 1C).

**Figure 2:**
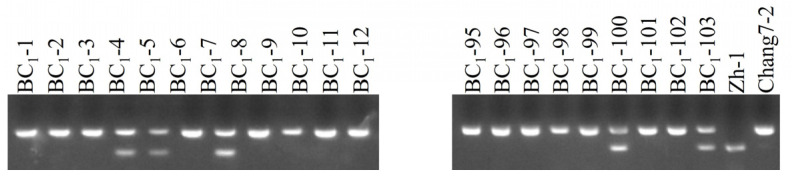
Agarose electrophoresis of InDel markers amplified from the 103 positive BC_1_ seedlings identified by PCR amplification of the transgene fragment.

Each of these 103 positive seedlings was transplanted into the field and backcrossed with the recurrent parent Chang 7-2. The results of InDel marker amplification and agarose gel electrophoresis indicated that the average recovery rate of the genetic background of the recurrent parent in the BC_1_ population was 74.80% (Figure 2 and Table S2). Among them, eighteen individual plants achieved a background recovery rate of over 80%, and the highest was 87.96% (plants BC_1_-27 and BC_1_-46) (Table 1). From these, seven plants (BC_1_-1, -5, -8, -35, -36, -47, and -83) with similar phenotypic traits to those of the recurrent parent Chang 7-2 were backcrossed to the recurrent parent Chang 7-2 to generate the BC_2_ lines. Through glufosinate spraying and PCR amplification of the transgenic AnVP1 gene, seventy positive individual plants were identified and then backcrossed with their recurrent parent Chang 7-2 again to reproduce BC_2_ lines.

The average recovery rate of the genetic background of the recurrent parent in the BC_2_ lines was 91.93% (Figure S3 and Table 2). Two BC_2_ plants, BC_2_-36-12 and BC_2_-5-15, with background recovery rates of 95.40% and 94.77%, respectively, and similar phenotypic traits to those of the recurrent parent Chang 7-2, were finally selected for self-pollination over two generations to reproduce BC_2_F_2_ lines. Two homozygous lines were identified among the BC_2_F_2_ lines. Their plant height, ear height, tassel branch number, ear length, ear diameter, and 100-kernel weight were non-significant compared to those of the recurrent parent Chang 7-2 (Table 3). Consequently, these two lines were self-pollinated to produce homozygous BC_2_F_3_ lines.

### 2.3. Stable Expression of Transgenic AnVP1 Gene

**Figure 3:**
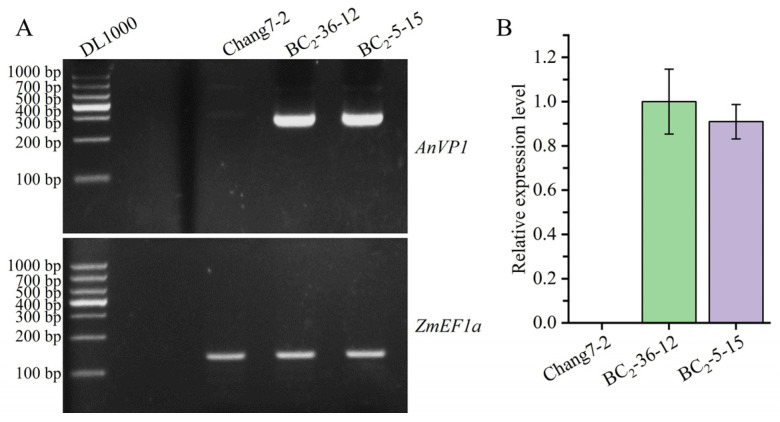
Stable expression of transgenic AnVP1 gene detected by RT-PCR (**A**) and RT-qPCR (**B**). The endogenous *ZmEFla* gene is used as a reference. Error bars represent the standard error.

The calibration curve and primer efficiency for RT-qPCR are shown in Figure S4. The results of RT-PCR and RT-qPCR indicated that the transgenic *AnVP1* gene was stably expressed in the two selected BC_2_F_3_ lines, BC_2_-36-12 and BC_2_-5-15, but not detected in their recurrent parent Chang 7-2. Their relative expression levels were several times higher than those of the internal reference gene *ZmEF1a* (Figure 3A,B).

### 2.4. Enhancement of Drought Tolerance

**Figure 4:**
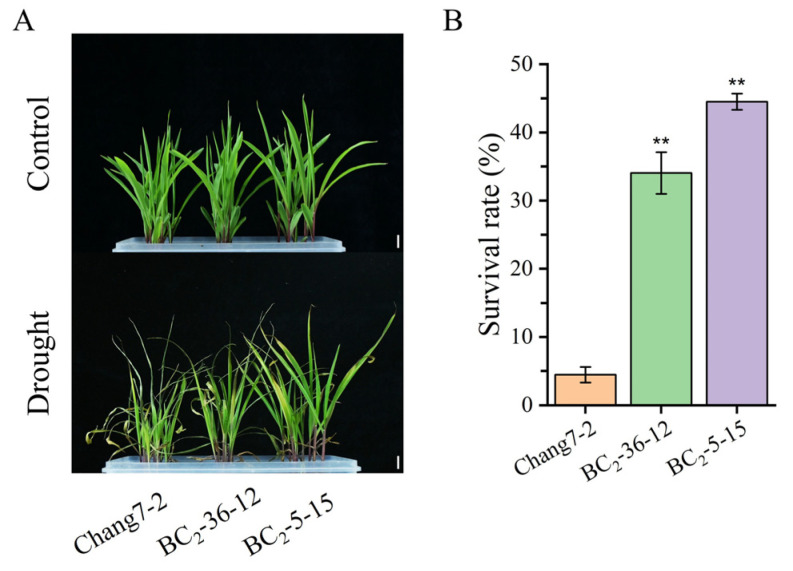
Analysis of drought resistance at the seedling stage: Representative photographs of maize seedlings before and after drought treatment (**A**). The survival rate after drought treatment (**B**). The double asterisk (**) indicates a significance at *p* ≤ 0.01. Error bars represent the standard error. The scale bar is 2 cm.

**Figure 5:**
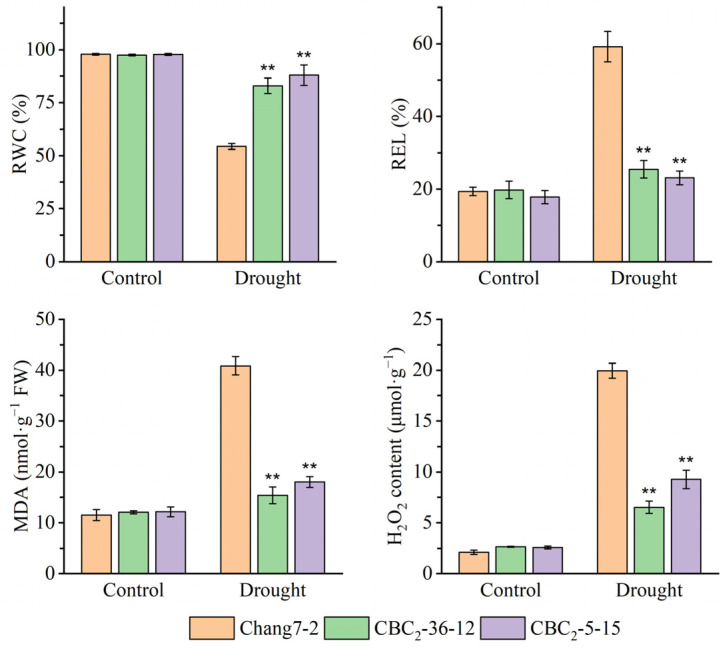
Measurement of RWC, REL, MDA, and H_2_O_2_ of the two BC_2_F_3_ lines before and after drought treatment at the seedling stage. The double asterisk (**) indicates a significance at *p* ≤ 0.01. Error bars represent the standard error.

Beginning on the third day following water deprivation, the seedlings of the recurrent parent Chang 7-2 commenced wilting. In contrast, the seedlings of the two BC_2_F_3_ lines BC_2_-36-12 and BC_2_-5-15 grew normally (Figure 4A). Their survival percentages were significantly different at *p* ≤ 0.01 (Figure 4B). When compared to the recurrent parent Chang 7-2, the RWC of the two BC_2_F_3_ lines, BC_2_-36-12 and BC_2_-5-15, was significantly higher, while their REL, MDA and H_2_O_2_ content were significantly lower at *p* ≤ 0.01 (Figure 5). These results clearly demonstrated that the transgenic *AnVP1* gene enhanced the drought tolerance of the recurrent parent Chang 7-2.

### 2.5. Field Phenotype of Drought Tolerance

**Figure 6:**
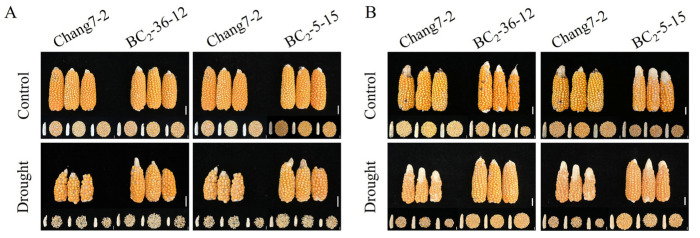
Ear phenotypes of the two BC_2_F_3_ lines under the two semi-arid and arid environments: (**A**): Gaotai; (**B**): Pucheng. The scale bar is 2 cm.

**Figure 7:**
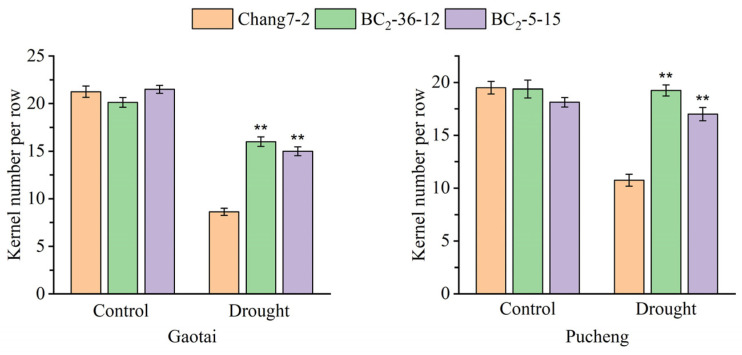
Kernel number per row of the two BC_2_F_3_ lines and their recurrent parent Chang 7-2 under the two semi-arid and arid environments. The double asterisk (**) indicates a significance at *p* ≤ 0.01. Error bars represent the standard error.

**Figure 8:**
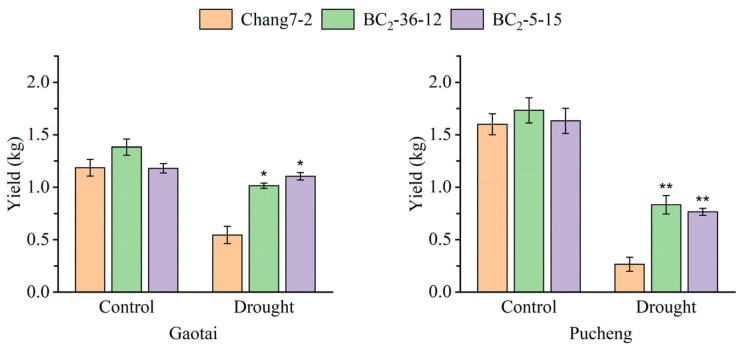
Yield of the two BC_2_F_3_ lines and their recurrent parent Chang 7-2 under the two semi-arid and arid environments. The asterisk (*) and double asterisk (**) indicates a significance at *p* ≤ 0.05 and *p* ≤ 0.01, respectively. Error bars represent the standard error.

**Figure 9:**
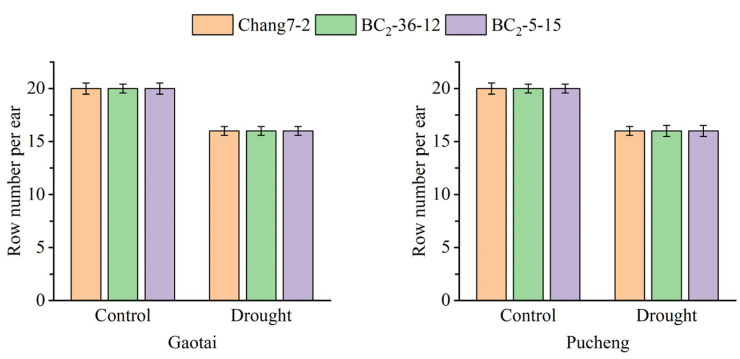
Row number per ear of the two BC_2_F_3_ lines and their recurrent parent Chang 7-2 under the two semi-arid and arid environments. Error bars represent the standard error.

**Figure 10:**
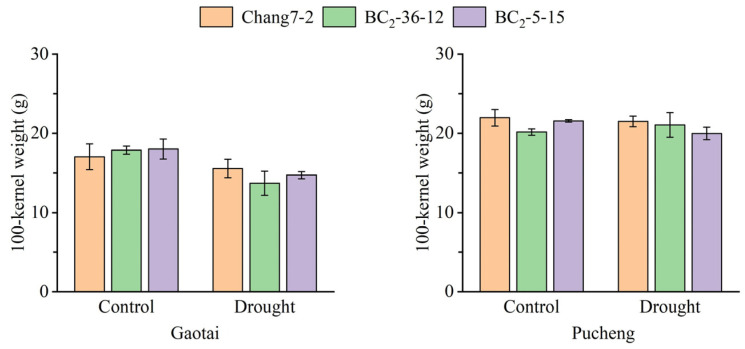
The 100-kernel weight of the two BC_2_F_3_ lines and their recurrent parent Chang 7-2 under the two semi-arid and arid environments. Error bars represent the standard error.

The results of the field phenotyping at Gaotai and Pucheng were nearly identical (Figure 6). Under normal irrigation conditions, there were no significant differences in the row number per ear, 100-kernel weight, kernel number per row, and kernel weight per plant between the two BC_2_F_3_ lines and their recurrent parent Chang 7-2 (Figure 7, Figure 8, Figure 9 and Figure 10). However, under drought stress, the kernel number per row and yield were significantly different between the two BC_2_F_3_ lines and their recurrent parent Chang 7-2 (Figure 7 and Figure 8), while the differences in row number per ear and 100-kernel weight remained non-significant (Figure 9 and Figure 10). These results indicated that the backcrossing of the transgenic AnVP1 gene improved the drought tolerance of yield mainly by increasing the kernel number per row.

## 3. Discussion

Drought stress is a major stimulus during agricultural production and severely restricts maize growth and yield [52,53]. Hence, improving maize drought tolerance is of significance for food security and economic development in the world. In our previous study, transgenic maize with the *AnVP1* gene was generated and exhibited an enhancement of drought tolerance [24]. Therefore, in the present study, the transgenic line with *AnVP1* was used to backcross into the recurrent parent Chang 7-2, one of the elite parents of Zhengdan 958, to produce improved Chang 7-2 with higher drought tolerance.

From the 1749 InDel sites between the genome sequences of inbred lines Chang 7-2 and Zh-1, 103 InDel markers with amplified lengths of 100–200 bp and roughly evenly distributed on the 10 chromosomes of the maize genome were screened and evaluated to apply to marker-assisted backcrossing of the *AnVP1* gene from the homozygous transgenic Zh-1 lines to recurrent parent Chang 7-2 (Tables S1 and S2, Figures S1 and S2). However, the number of the screened InDel markers were a bit too few for the second long chromosome 2. Since there were few differences between Chang 7-2 and Zh-1 in this region, no suitable markers were found. Moreover, the gene insertion site of the L10 lines was on chromosome 8 of the genome, so it did not affect the selection of the recurrent parent background recovery rate. After two generations of foreground selection by glufosinate spraying, the detection of CP4 EPSP MAb1 strips, and the PCR amplification of the transgenic *AnVP1* gene (Figure 1A), along with the similarity of agronomic traits to the recurrent parent, and background selection assisted by InDel markers (Figure 2 and Figure S3 and Table 3), the transgenic *AnVP1* gene was homozygous in the BC_2_ lines. The average recovery rate of the genetic background of the recurrent parent reached 74.80% in the BC_1_ population (Table 1 and Table S2) and 91.93% in the BC_2_ population (Table 2), respectively. This progress was two or three generations shorter than that of homozygous lines obtained in the BC_4_ and BC_5_ populations in the STMS marker-assisted backcrossing of the transgenic *GR2* gene from donor parent to recurrent parent in the improvement of ‘Golden Rice’. Their background recovery rate did not exceed over 95% until the BC_4_ and BC_5_ generations [34,36]. The average background recovery rate of the 70 BC_2_ lines (91.92%, Table 2) was even 9.11% higher than that (82.81%) of the BC_2_F_3_ lines of SSR marker-assisted backcrossing of the transgenic *cry1Ac* gene for pod borer resistance in chickpea [39], and it was comparable to that (90.24%-92.42%) of the BC_2_F_3_ lines of SSR marker-assisted backcrossing of the transgenic *β*-carotene gene *crtRB1* for the biofortification of provitamin A carotenoids [42] and that (92.89%) of the BC_3_F_4_ lines SSR and InDel marker-assisted backcrossing of the transgenic *Pi2*, *Pi46*, and *Pita* genes for pyramiding the improvement of rice blast resistance [54].

Both the results of RT-PCR and RT-qPCR indicated the stable expression of the transgenic *AnVP1* gene in the two ultimately selected BC_2_F_3_ lines BC_2_-36-12 and BC_2_-5-15 (Figure 3), similar to that in the donor parent L10 transformed from Zh-1 [24]. The drought tolerance of these two BC_2_F_3_ lines was significantly improved compared to their recurrent parent Chang 7-2, as revealed by their wilting phenotypes and seedling survival rates (Figure 4). This improvement was related to the enhancement of water retention ability, as indicated by RWC and the reduction in damage, as shown by the accumulation of REL, MDA, and H_2_O_2_ under drought stress (Figure 5). The result of field evaluation in two semi-arid and arid environments indicated that the recurrent parent Chang 7-2 had its drought tolerance improved through the backcross breeding of the transgenic *AnVP1* gene (Figure 6, Figure 7, Figure 8, Figure 9 and Figure 10). According to the regulations on the safety management of agricultural genetically modified organisms (GMOs) in China, after further evaluation of GMO safety, the improved Chang 7-2 is suggested to be crossed with the other parent Zheng 58 to produce a transgenic commercial hybrid Zhengdan 958, to enhance the drought tolerance of this important hybrid.

## 4. Materials and Methods

### 4.1. Identification of Length Variation Sites

The raw data of genome resequencing of Zh-1 and the genome sequences of Chang 7-2 [24,55] were filtered using the FASTP preprocessor (https://github.com/OpenGene/fastp, accessed on 15 March 2020 [56]). The clean reads were aligned to the reference genome sequences of maize inbred line B73 (https://download.maizegdb.org, accessed on 17 March 2020 [57]) by BWA software (http://manpages.ubuntu.com/, accessed on 17 March 2020 [58]). The potentially duplicated sequences resulting from PCR amplification in Illumina sequencing were removed by Markduplicate software of Picard toolkit (http://broadinstitute.github.io/picard/, accessed on 19 March 2020 [59]). The length variation sites among the genome sequences of Zh-1 and Chang 7-2 were identified using the HaplotypeCaller software in the GATK toolkit (https://github.com/RRafiee/GenomeAnalysisToolkit, accessed on 21 April 2020 [60]) and then filtered for genotype quality (GQ > 30) using VCFtools software (http://vcftools.sourceforge.net/, accessed on 26 April 2020 [61]).

### 4.2. Design, Screening, and Evaluation of InDel Markers

The length variation sites ranging from 30 to 50 bp were screened using a Perl script. PCR primers were designed using Primer v 6.0 (http://primerinc.com/, accessed on 26 May 2020) to amplify sequences of 120–150 bp flanking to identified length variation sites. The resulting sequences were aligned to the reference genome sequences of maize inbred line B73 using Blast software v2.9.0 (https://blast.ncbi.nlm.nih.gov/Blast.cgi, accessed on 26 May 2020). Sequences with one or more repeats and a G/C base ratio less than 55% or more than 60% were filtered out. Additionally, sequences were screened for the following conditions: having the same as B73 with base-mismatch ≤ 2, being 1 bp different from B73 with a base-mismatch ≤ 1, or being 2 bp different from B73 with no mismatch base. Based on the result of the simulative selection conducted by Visscher et al. [62], 103 InDel markers with a distance of about 20 cM and evenly distributed in the maize genome were screened by a Perl script. PCR primers were designed and used to amplify the screened InDel markers from the genomic DNA of inbred lines Zh-1 and Chang 7-2, aiming to evaluate their polymorphism between these two lines. The PCR reaction was conducted in the Eppendorf 6331 Nexus Gradient MasterCycler Thermal Cycler (Eppendorf, Hamburg, Germany) with 2 × Rapid Taq Master Mix (Vazyme, Nanjing, China). A touch-down temperature protocol was 95 °C for 3 m, 10 cycles of 95 °C for 15 s, 65 °C (reduced by 1 °C per cycle) for 15 s, and 72 °C for 10 s, then followed by 20 cycles of 95 °C for 15 s, 55 °C for 15 s, and 72 °C for 10 s.

### 4.3. Foreground Selection

One (L10) of the five homozygous T_8_ lines with enhanced drought tolerance was used as a donor parent to cross with recurrent parent Chang 7-2. The F_1_ generation and the selected plants from subsequent backcrossing generations were backcrossed to the recurrent parent. At the three-leaf stage of each backcrossing generation, the seedlings were sprayed with 0.7‰ glufosinate solution. Five days later, withered seedlings were identified as negative and removed. The segregation ratio of positive to negative seedlings was investigated, and its consistency with the theoretical ratio of 1:1 was tested by the χ^2^ test. At the five-leaf stage, the leaf sample was punched from each of the positive seedlings and lines. The samples were used for the detection of the markerBar gene on the expression vector using CP4 EPSP MAb1 strips (Aochuang Biotech, Chengdu, China [63]) and for PCR amplification of an 865 bp fragment spanning the Ubi promoter and the transgenic AnVP1 gene with primers (AnVP1-F: 5′-CTGCGCTGTCATTGGAATCG-3′/AnVP1-R: 5′-GGCACAGGAAGACTCAGCAT-3′). The reaction system and instrument were the same as described above. The three-step temperature cycle was 95 °C for 3 min and 35 cycles of 95 °C for 15 s, 60 °C for 30 s, and 72 °C for 15 s.

### 4.4. InDel Marker-Assisted Background Selection

Leaf samples were collected from the positive seedlings and lines selected in the foreground screening and used for the genomic DNA extraction. The primers of the evaluated InDel markers were used for polymorphism detection as described above. The numbers of specific bands corresponding to the recurrent parent and heterozygous bands was recorded to calculate the background recovery rate using the following formula:

$$\;\mathrm{Rg}=\frac{I+S}{2\;\times \;I}\;\times \;100\%$$

where Rg represents the recovery rate of the recurrent parent’s genetic background in backcrossing generation g, I is the total number of InDel markers used, and S is the number of specific bands matching the recurrent parent.

Individual plants or backcrossing lines with a recurrent parent genetic background recovery rate of more than 80% and phenotypic traits similar to those of their recurrent parent were self-pollinated to produce the next generation. When their recovery rate exceeded 90% and phenotypic traits were close to those of their recurrent parent, the individual plants or backcrossing lines were self-pollinated for two generations. The homozygous lines were identified from the BC_n_F_2_ lines as above. Agronomic traits such as plant height, ear height, tassel branch number, ear length, ear diameter, and 100-kernel weight were investigated. These lines then self-pollinated to generate homozygous BC_n_F_3_ lines.

### 4.5. RT-PCR and RT-qPCR

Leaf samples were punched from the selected homozygous BC_n_F_2_ plants and Chang 7-2 under optimal conditions. These samples were then used to isolate total RNA using the AFTSpin Universal Plant Fast RNA Extraction Kit (ABclonal, Wuhan, China). Each of these RNA samples were reverse-transcribed into cDNA using the ABScript III RT Master Mix for qPCR with gDNA Remover (ABclonal). The specific primers of RT-PCR and RT-qPCR for amplification of the fragments of the transgenic AnVP1 gene (AnVP1-QF: 5′-GCTTGATGGAGGGAACTGCT-3′/AnVP1-QR: 5′-CCTTTGGGACCAAGGCTTCT-3′) and the reference gene ZmEF1a (ZmEF1a-F: 5′-TGGGCCTACTGGTCTTACTACTGA-3′/ZmEF1a-R: 5′-ACATACCCACGCTTCAGATCCT-3′ [64]) were designed using the online software Primer-BLAST (https://www.ncbi.nlm.nih.gov/tools/primer-blast/index.cgi?LINK_LOC=BlastHome, accessed on 12 July 2022) and synthesized at Sangon Biotech (Shanghai, China). RT-PCR was performed using the above cDNA as the template and TaKaRa Taq™ enzymes (TaKaRa, Kusatsu, Japan). The RT-qPCR was performed in the Bio-Rad CFX96^TM^ Real-Time PCR system with 2 × Universal SYBR Green Fast qPCR Mix (ABclonal). The relative expression level was calculated and normalized using the 2^−ΔΔCt^ method of the Bio-Rad CFX Maestro 1.1 (https://www.bio-chain.com/Bio-Bad-Product-Details.html?product_id=415, accessed on 27 July 2022). The average relative expression levels of the transgenic AnVP1 gene of the four technical replicates and the three biological replicates were calculated by comparison with the relative expression level of the internal reference gene ZmEF1a [65].

### 4.6. Pot Evaluation of Drought Tolerance

The seeds of the homozygous BC_2_F_3_ lines and Chang 7-2 were incubated in seedling trays under the conditions of 28 °C, 14 h of light, and 22 °C, 10 h of dark. At the three-leaf stage, ten uniform seedlings of each were transplanted into the same culture box (36 cm in length × 24 cm in width × 11 cm in depth) containing nutrient soil, with three replicates. At the five-leaf stage, the first leaf of each seedling was sampled, and, then, watering was ceased until obvious wilting occurred. After photographing and sampling the second leaf of each seedling, drought stress was continued until most seedlings of the recurrent parent Chang 7-2 withered severely. After determining the survival percentage, the leaf samples taken before and after water cessation were used to measure RWC, REL, and MDA content as described by Yu et al. [20]. Additionally, the H_2_O_2_ content was measured using an H_2_O_2_ content assay kit (Solarbio, Beijing, China).

### 4.7. Field Phenotyping of Drought Tolerance

The evaluated lines in the above pot experiment were further phenotyped for drought tolerance in field under arid (annual precipitation 146.7 mm) and semi-arid (annual precipitation 519.9 mm) environments at Gaotai County (39°16′ N, 99°55′ E,), Zhangye City, Gansu Province, and Pucheng County (35°10′ N, 109°20′ E), Weinan City, Shaanxi province of Northwest China. Normal irrigation and drought stress were designed as the two whole plots, and the evaluated lines were designed as the split plots of a split-plot experiment of three replicates. Three-leaf seedlings of each of the evaluated lines were incubated in the seedbed and then transplanted into the split plots. Each split plot contained two rows that were 3 m long with row space of 0.6 m, plant space of 0.25 m, and density of 59,400 plants/hectare. The ears were harvested from the plants of each split-plot except the plants at the ends of each row, air-dried to the standard moisture content of maize grains (GB 1353-1999 [66]), investigated for row number per ear, kernel number per row, 100-kernel weight, and yield of each split-plot.

### 4.8. Statistical Analysis

All data were analyzed using IBM-SPSS v28.0.0.0 (https://www.ibm.com/cn-zh/spss, accessed on 25 November 2024). The figures were created using OriginPro v9.8.0.200 (https://adobe.99vl.cn, accessed on 30 November 2024).

## 5. Conclusions

In conclusion, two BC_2_F_3_ lines, BC_2_-36-12 and BC_2_-5-15, were screened. These lines demonstrated enhanced drought tolerance, characterized by minimal wilting phenotypes, higher survival rates and RWC, lower REL, MDA, and H_2_O_2_, as well as higher yields under drought conditions. This study indicates that the improved Chang 7-2 can be hybridized with the other parent, Zheng 58, to generate a transgenic commercial hybrid, Zhengdan 958. This study also reveals that transgenic genes from transgenic recipients can be readily backcrossed into elite varieties or parents of commercial hybrids with the assistance of InDel markers.

## Figures and Tables

**Table 1:** Background recovery rates of 18 BC_1_ plants between Chang 7-2 and L10.

Plant	Recovery Rate	Plant	Recovery Rate	Plant	Recovery Rate
BC_1_-1	86.11%	BC_1_-34	85.18%	BC_1_-47	85.19%
BC_1_-5	87.03%	BC_1_-35	80.55%	BC_1_-53	81.48%
BC_1_-6	81.48%	BC_1_-36	81.48%	BC_1_-54	80.55%
BC_1_-8	83.33%	BC_1_-41	86.11%	BC_1_-83	81.48%
BC_1_-20	81.48%	BC_1_-45	83.33%	BC_1_-95	85.18%
BC_1_-27	87.96%	BC_1_-46	87.96%	BC_1_-101	84.26%

**Table 2:** Background recovery rate of 70 BC_2_ plants between Chang 7-2 and L10.

Plant	Recovery Rate	Plant	Recovery Rate	Plant	Recovery Rate
BC_2_-1-1	92.53%	BC_2_-8-6	94.25%	BC_2_-36-10	92.44%
BC_2_-1-2	91.95%	BC_2_-8-8	92.53%	BC_2_-36-11	92.35%
BC_2_-1-3	93.10%	BC_2_-8-9	94.25%	BC_2_-36-12	95.40%
BC_2_-1-4	90.80%	BC_2_-8-10	93.10%	BC_2_-47-1	92.44%
BC_2_-1-5	93.60%	BC_2_-8-11	91.95%	BC_2_-47-2	93.53%
BC_2_-1-6	91.95%	BC_2_-8-12	89.53%	BC_2_-47-3	90.80%
BC_2_-1-7	93.68%	BC_2_-8-13	93.68%	BC_2_-47-4	94.25%
BC_2_-1-8	92.44%	BC_2_-35-1	90.23%	BC_2_-47-5	90.23%
BC_2_-5-1	90.70%	BC_2_-35-2	89.53%	BC_2_-47-6	89.66%
BC_2_-5-2	93.10%	BC_2_-35-3	88.51%	BC_2_-47-7	94.25%
BC_2_-5-3	93.60%	BC_2_-35-4	91.38%	BC_2_-47-8	93.10%
BC_2_-5-4	90.80%	BC_2_-35-5	90.80%	BC_2_-83-1	94.19%
BC_2_-5-5	94.25%	BC_2_-35-6	90.23%	BC_2_-83-2	89.08%
BC_2_-5-6	91.38%	BC_2_-35-7	89.08%	BC_2_-83-4	91.38%
BC_2_-5-7	91.95%	BC_2_-35-8	90.23%	BC_2_-83-5	93.10%
BC_2_-5-8	87.93%	BC_2_-35-9	91.95%	BC_2_-83-6	93.10%
BC_2_-5-9	93.68%	BC_2_-36-1	93.10%	BC_2_-83-7	92.77%
BC_2_-5-10	91.86%	BC_2_-36-2	90.80%	BC_2_-83-8	90.23%
BC_2_-5-12	89.08%	BC_2_-36-3	94.25%	BC_2_-83-9	90.80%
BC_2_-5-15	94.77%	BC_2_-36-4	90.23%	BC_2_-83-10	89.66%
BC_2_-8-1	90.80%	BC_2_-36-5	91.95%	BC_2_-83-11	93.02%
BC_2_-8-2	92.53%	BC_2_-36-7	92.44%	BC_2_-83-12	92.94%
BC_2_-8-3	93.68%	BC_2_-36-8	90.36%		
BC_2_-8-4	93.10%	BC_2_-36-9	88.37%		

**Table 3:** Agronomic traits of the two BC_2_F_3_ lines.

Plant	Plant Height (cm)	Ear Height (cm)	Tassel Branch Number	Ear Length (cm)	Ear Diameter (cm)	100 Kernel Weight (g)
Chang 7-2	174	72	12	9.5	4.1	22.4
BC_2_-36-12	174	72	12	9.5	4.3	22.0
BC_2_-5-15	172	73	13	9.7	4.2	22.6

## Data Availability

Data are contained within the article and Supplementary Materials.

## Supplementary Materials

The following supporting information can be downloaded at https://www.mdpi.com/article/10.3390/plants14060926/s1, Figure S1. Distribution of 103 polymorphic InDel markers between Chang 7-2 and Zh-1 on the chromosomes. Figure S2. Polymorphism of 103 InDel markers between Chang 7-2 and Zh-1. Figure S3. InDel molecular marker background selection for the BC_2_ population. Figure S4. Standard curves of quantitative primers for the *AnVP1* (A) and *ZmEF1a* (B) genes. Table S1. 103 InDel markers and their PCR primers between Chang 7-2 and Zh-1. Table S2. The background recovery rate of 103 BC_1_ plants between Chang 7-2 and L10.
