# Supplementary material for: Marker-Assisted Backcross Breeding of Drought-Tolerant Maize Lines Transformed by Vacuolar H+-Pyrophosphatase Gene (AnVP1) from Ammopiptanthus nanus
Source: Plants (Basel). 2025 Mar 15;14(6):926. doi: 10.3390/plants14060926 (PMC11945134; doi:10.3390/plants14060926)
Supplement: Supplementary file 1 [file plants-14-00926-s001.zip › plants-3506515-supplementary.pdf]

# Supplementary Material

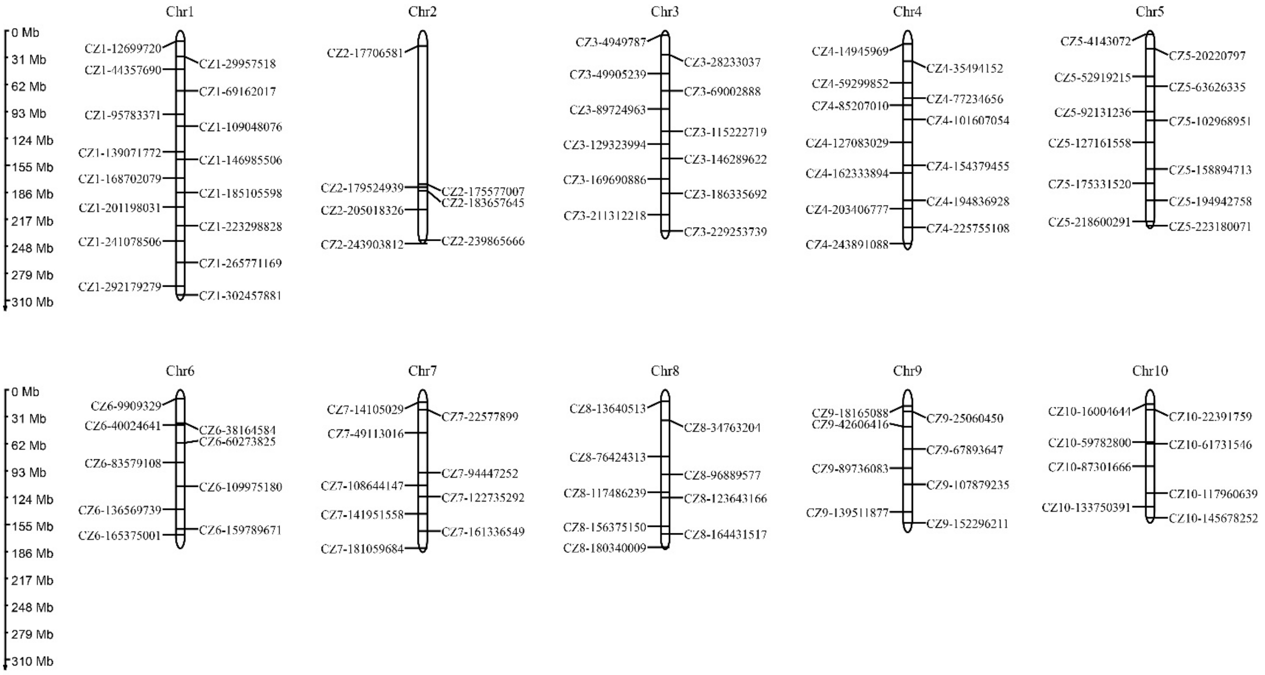


**Figure S1** Distribution of 103 polymorphic InDel markers between Chang 7-2 and Zh-1 on the chromosomes.

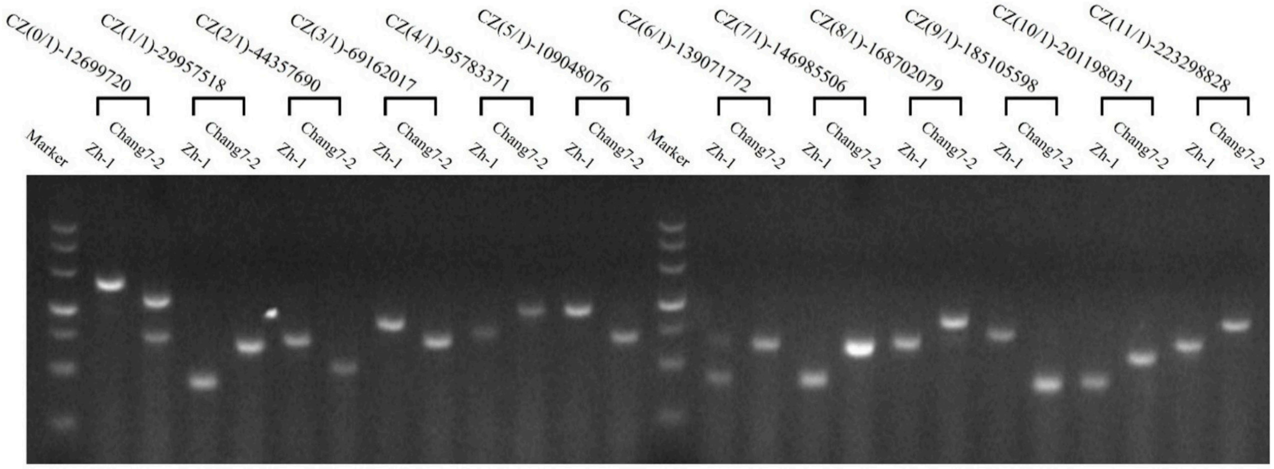

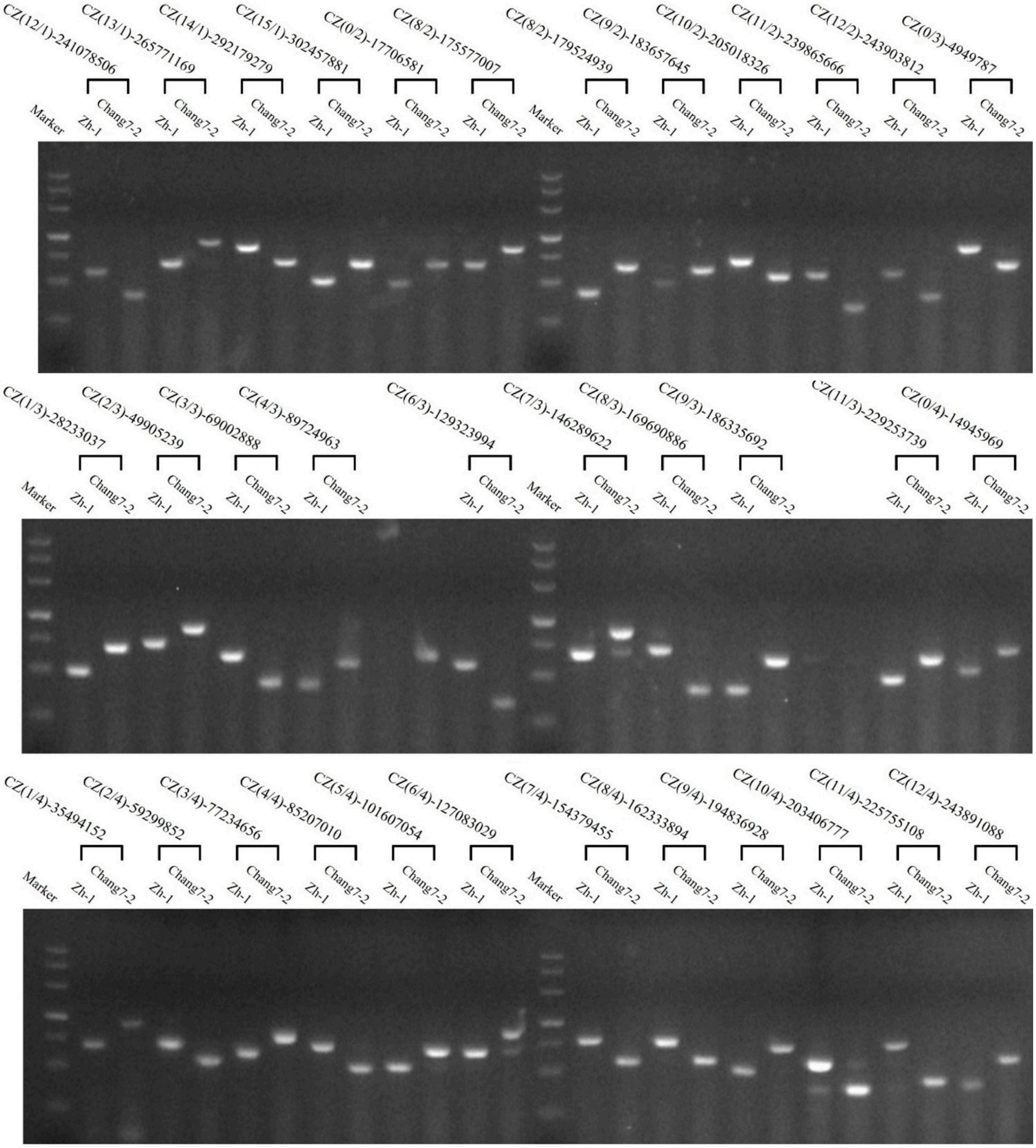
CZ(12/1)-241078506
CZ(13/1)-265771169
CZ(14/1)-292179279
CZ(15/1)-302457881
CZ(0/2)-17706581
CZ(8/2)-175577007
CZ(8/2)-179524939
CZ(9/2)-183657645
CZ(10/2)-205018326
CZ(11/2)-239865666
CZ(12/2)-243903812
CZ(0/3)-4949787
Marker
Zh-1
Chang7-2
CZ(1/3)-28233037
CZ(2/3)-49905239
CZ(3/3)-69002888
CZ(4/3)-89724963
CZ(6/3)-129323994
CZ(7/3)-146289622
CZ(8/3)-169690886
CZ(9/3)-186335692
CZ(11/3)-229253739
CZ(0/4)-14945969
Marker
Zh-1
Chang7-2
CZ(1/4)-35494152
CZ(2/4)-59299852
CZ(3/4)-77234656
CZ(4/4)-85207010
CZ(5/4)-101607054
CZ(6/4)-127083029
CZ(7/4)-154379455
CZ(8/4)-162333894
CZ(9/4)-194836928
CZ(10/4)-203406777
CZ(11/4)-225755108
CZ(12/4)-243891088
Marker
Zh-1
Chang7-2

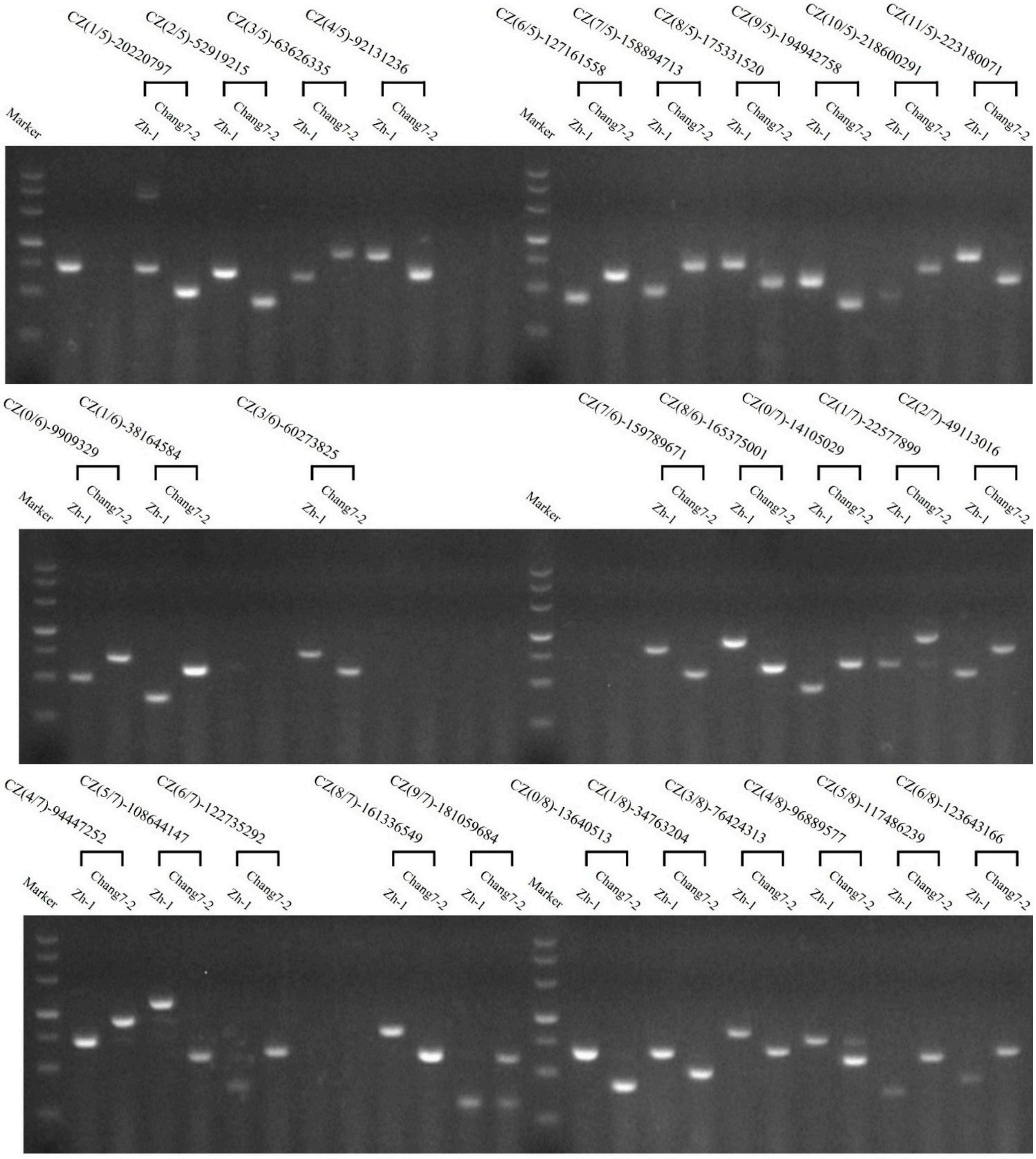
CZ(1/5)-20220797
CZ(2/5)-52919215
CZ(3/5)-63626335
CZ(4/5)-92131236
CZ(6/5)-127161558
CZ(7/5)-158894713
CZ(8/5)-175331520
CZ(9/5)-194942758
CZ(10/5)-218600291
CZ(11/5)-223180071
Marker
Zh-1
Chang7-2
CZ(0/6)-9909329
CZ(1/6)-38164584
CZ(3/6)-60273825
CZ(7/6)-159789671
CZ(8/6)-165375001
CZ(0/7)-14105029
CZ(1/7)-22577899
CZ(2/7)-49113016
CZ(4/7)-94447252
CZ(5/7)-108644147
CZ(6/7)-122735292
CZ(8/7)-161336549
CZ(9/7)-181059684
CZ(0/8)-13640513
CZ(1/8)-34763204
CZ(3/8)-76424313
CZ(4/8)-96889577
CZ(5/8)-117486239
CZ(6/8)-123643166

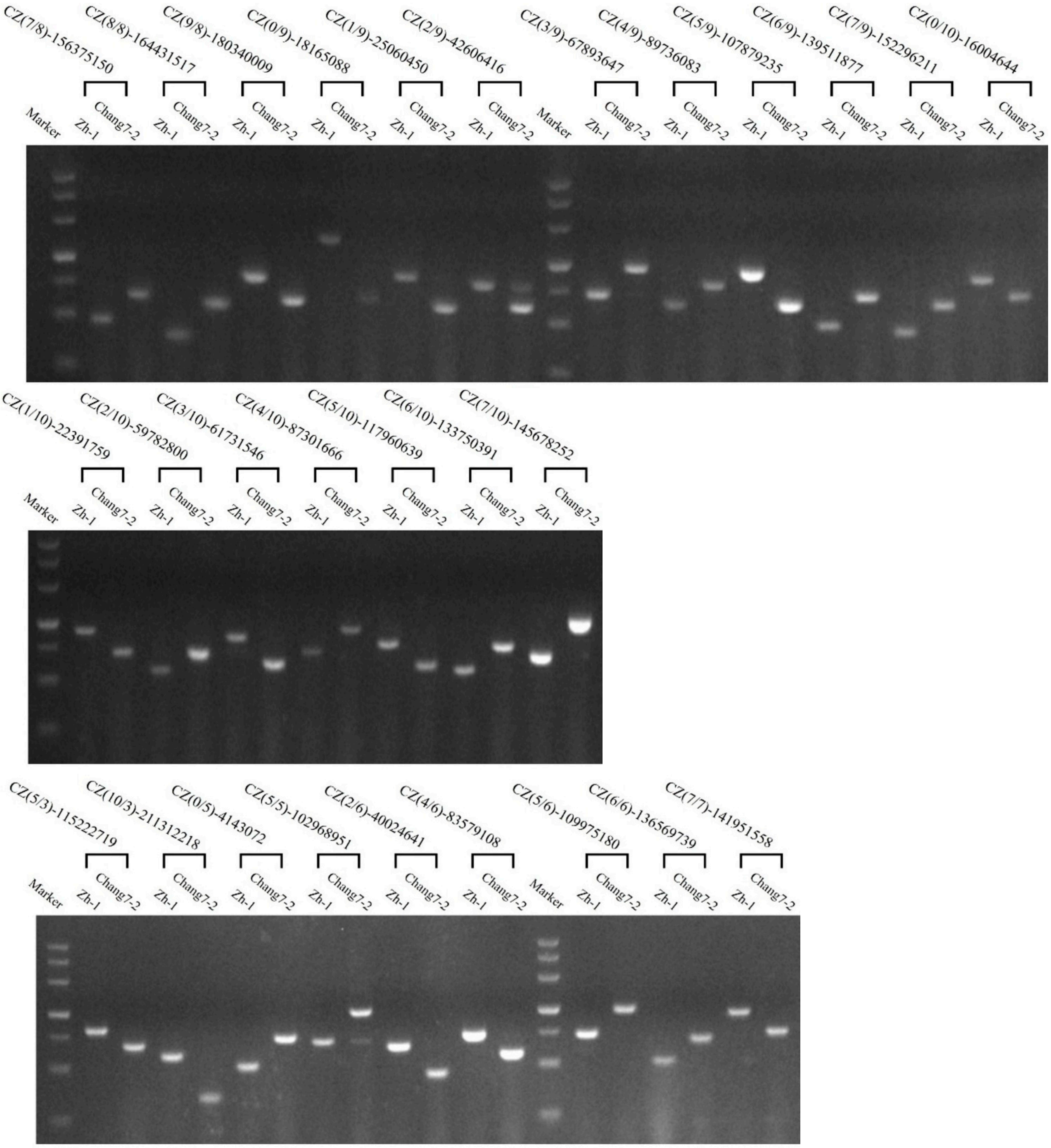


**Figure S2** Polymorphism of 103 InDel markers between Chang 7-2 and Zh-1.

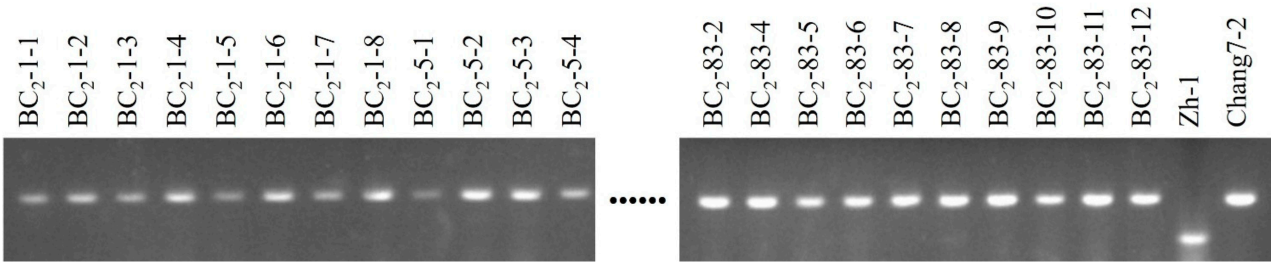


**Figure S3** InDel molecular marker background selection for the $BC_2$ population.

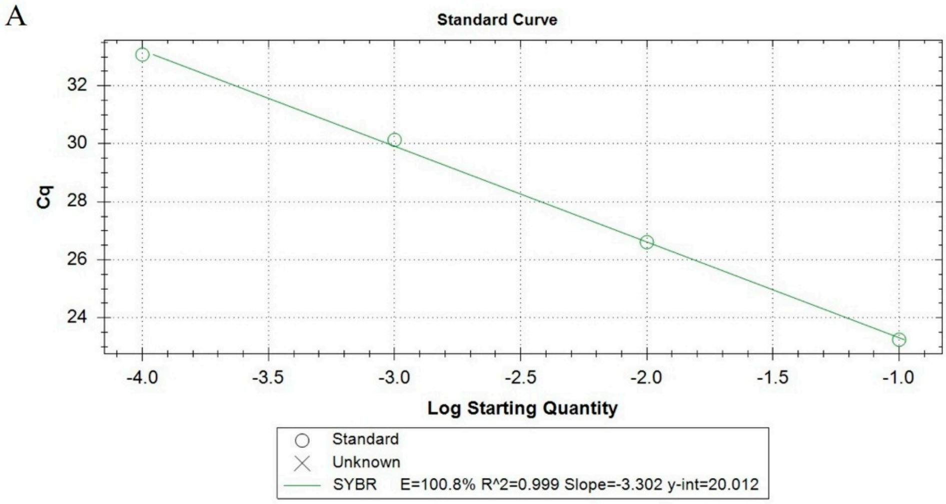


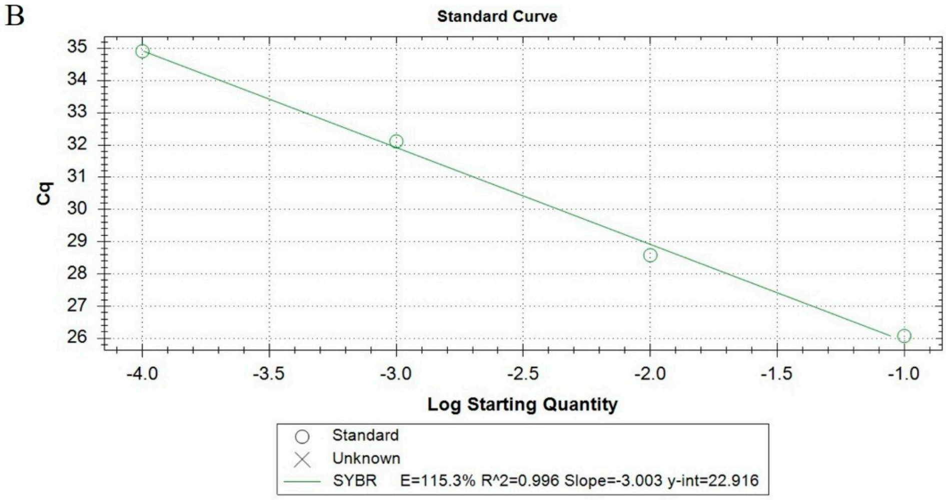


**Figure S4** Standard curves of quantitative primers for the *AnVP1* (A) and *ZmEF1a* (B) genes.

**Table S1** 103 InDel markers and their PCR primers between Chang 7-2 and Zh-1

| Marker | Primer sequence |
|---|---|
| CZ(0/1)12699720 | 5′-CAAAGCGCCAACTACGTTTT-3′/5′-TCCACCCTTACTGTAGCAGC-3′ |
| CZ(1/1)29957518 | 5′-CTGAACTCTGCAACGGCA-3′/5′-GTGTCCTGCAGCCTGTGAT-3′ |
| CZ(2/1)44357690 | 5′-TTGCAGCTGACCACAACC-3′/5′-AAAAGCCGGAGGATGAGC-3′ |
| CZ(3/1)69162017 | 5′-TTCGCAACATGAAGGGGT-3′/5′-AACCCACACGGAGCAGAT-3′ |
| CZ(4/1)95783371 | 5′-GCCCCAAAACACCAATTC-3′/5′-TCCACCACCACCGTGATA-3′ |
| CZ(5/1)109048076 | 5′-CTTCTCTCCGTTTCCCCTG-3′/5′-CCGCCAGCATCAATTTTT-3′ |
| CZ(6/1)139071772 | 5′-AACCAAAGCTCATTCACCG-3′/5′-TGGCCAATTGATGCTGAA-3′ |
| CZ(7/1)146985506 | 5′-ACACCGGTTGAGTTTGCC-3′/5′-TTCCATCGTTTCAAGACTCGT-3′ |
| CZ(8/1)168702079 | 5′-TGGGCAGAAACAGAAGCAG-3′/5′-TGTCAAGTTGCAACATCGTG-3′ |
| CZ(9/1)185105598 | 5′-TGCAACGCGCTTTAATTC-3′/5′-GGCAAAAGGCAAACTGCT-3′ |
| CZ(10/1)201198031 | 5′-CAACATACGTCCCTGGCA-3′/5′-CGAATACAATACGCAGCGG-3′ |
| CZ(11/1)223298828 | 5′-ACACGGCAAATGCCTGAC-3′/5′-TTCGTTTCAGGGTGCCAT-3′ |
| CZ(12/1)241078506 | 5′-ACTGACAGTTGCCACCGAG-3′/5′-CGCATGAGTGACCAAACG-3′ |
| CZ(13/1)265771169 | 5′-CGCAAACTGACGACGATG-3′/5′-TTGATGCGTGCGTTCTTC-3′ |
| CZ(14/1)292179279 | 5′-GGGGCGAGCATTATTCAA-3′/5′-GCACTTTCCAGTCCGCAA-3′ |
| CZ(15/1)302457881 | 5′-ACTGCCTGTCGCCATCTC-3′/5′-CAACTCGCATTGGTTGGC-3′ |
| CZ(0/2)17706581 | 5′-TTCCAGACATTGCAGGGG-3′/5′-GCTTCAACGTCTTCCCCA-3′ |
| CZ(8/2)175577007 | 5′-CACGTTGGACCAGGGTTT-3′/5′-AGCTTGCAAATCTGTCCCC-3′ |
| CZ(8/2)179524939 | 5′-AAAGCCGAATTCATGCGA-3′/5′-GCGAATGCAAATGGTGC-3′ |
| CZ(9/2)183657645 | 5′-ATCGCGCAGGAATAATGG-3′/5′-TGACGGCGTGCTATCAAC-3′ |
| CZ(10/2)205018326 | 5′-TTGAAACTGCGAAAGGGC-3′/5′-CGGTCCAACAAGAGGGTG-3′ |
| CZ(11/2)239865666 | 5′-TGAGACTCAGATTCCCACC-3′/5′-CTGAGATGTGCTGTGCGG-3′ |
| CZ(12/2)243903812 | 5′-TTGGTTATGCATCGTGCG-3′/5′-ATCCATTTTCACGGGCAAT-3′ |
| CZ(0/3)4949787 | 5′-CTTTTTCGTCGTGCCCAG-3′/5′-CATGGTAGCGAAGGCGAA-3′ |
| CZ(1/3)28233037 | 5′-AGAGTTGCTGGAGAAGCTGG-3′/5′-AATACTGCAACGGCCCAA-3′ |
| CZ(2/3)49905239 | 5′-TTGACGGTGGTGGTCAGA-3′/5′-TTGCGAGATGGAGGTGCT-3′ |
| CZ(3/3)69002888 | 5′-ATCTCTGCGGGGTTTGCT-3′/5′-AAAAGCCCCTCTTGCCAT-3′ |
| CZ(4/3)89724963 | 5′-CAGCCAGCCAAAAAGTGC-3′/5′-TTTGGATGTCGGCAGGAT-3′ |
| CZ(5/3)115222719 | 5′-TCATTCTGTGGGTGGATGG-3′/5′-TCGTGTGGGCATGACATT-3′ |
| CZ(6/3)129323994 | 5′-TCAGCGGAGAACACATCG-3′/5′-TGAACAGCCTGTGGTTCG-3′ |
| CZ(7/3)146289622 | 5′-AATGGTCGTCGTCTTGCG-3′/5′-ACGACGAACGCTGACACA-3′ |
| CZ(8/3)169690886 | 5′-TGGATCGTCACCTGCCA-3′/5′-CGACTCTGTTCTGCCTGTGA-3′ |
| CZ(9/3)186335692 | 5′-GGATGTGACTTCAGGAGCC-3′/5′-GCCCTGTAAAAGCATCCG-3′ |
| CZ(10/3)211312218 | 5′-CCCCGATCGATATTCCAG-3′/5′-CGATGTCAATGACTCAGCG-3′ |
| CZ(11/3)229253739 | 5′-TGTACTTGTGTGCTCCTGCC-3′/5′-ACGCGCACCTATATTGCAG-3′ |
| CZ(0/4)14945969 | 5′-ACAGATGCCGCTCGAAGT-3′/5′-TTAGCGGCCAATTTCCTTT-3′ |
| CZ(1/4)35494152 | 5′-CCACGAGTTGAACCATGC-3′/5′-GGCCCAATAGATCCAGCA-3′ |
| CZ(2/4)59299852 | 5′-AATTACGCGGTCCACACG-3′/5′-GAAGCAGATGCACTGGCA-3′ |
| CZ(3/4)77234656 | 5′-CCACCACGTGCATGGAT-3′/5′-AGCATTGAATGAGCGAAGTG-3′ |
| CZ(4/4)85207010 | 5′-TGGGTCCCTCGAACTTGA-3′/5′-CAAAAGCGTTCGAAACCG-3′ |
| CZ(5/4)101607054 | 5′-CACGAGGAGTTGAGACCGA-3′/5′-ACCACAACGAGAACACATCAT-3′ |
| CZ(6/4)127083029 | 5′-CTCCCATTGCCCCTCTCT-3′/5′-TCGGCGAGATCCGATTAC-3′ |
| CZ(7/4)154379455 | 5′-CGTCTGGCTTTCTGAGGC-3′/5′-CGGCACTTCCTATCGGTC-3′ |
| CZ(8/4)162333894 | 5′-CCAACTAGGGGTTTCACCA-3′/5′-ACGGGCATCGAGTTATTCC-3′ |
| CZ(9/4)194836928 | 5′-CGAATGCAGCAATAGCCA-3′/5′-CGTTGCAGGGGGAAATAA-3′ |

| | |
|---|---|
| CZ(10/4)203406777 | 5′-GAAAATAACCATCCAGTGCCG-3′/5′-TGGTTAAGCTTGCTGTCTCC-3′ |
| CZ(11/4)225755108 | 5′-CCTCACCACCATCATGAGTC-3′/5′-AGGTGCAACATCAGGATGAA-3′ |
| CZ(12/4)243891088 | 5′-TCCATCGCAAGAAAGTTGG-3′/5′-ATGGCCGTCAGTTTCACG-3′ |
| CZ(0/5)4143072 | 5′-CATTGTGCCGTGAAGACG-3′/5′-AAGCCGTCGTCAAAGGTT-3′ |
| CZ(1/5)20220797 | 5′-ATAGAGCTGGTGGTGCGTG-3′/5′-GGGTGAGGAGAAATGAGTGG-3′ |
| CZ(2/5)52919215 | 5′-GCTCTTTCTGGTCACACTGCT-3′/5′-TGTAGGCTTGCTGGTGGAG-3′ |
| CZ(3/5)63626335 | 5′-TGCACATACGCCTTCGAG-3′/5′-TTGATTTGTGCTTCCAGGC-3′ |
| CZ(4/5)92131236 | 5′-ATGACATTCCCCTCCGCT-3′/5′-CGTTGTTGGCCAGTATCCA-3′ |
| CZ(5/5)102968951 | 5′-CAACGGTGTCATTGGTTTG-3′/5′-CAGCTGCAGGGACATCAA-3′ |
| CZ(6/5)127161558 | 5′-GGATGTTGCAGCGCTTTT-3′/5′-CCTGAGGCACACGCTTCT-3′ |
| CZ(7/5)158894713 | 5′-TCCTACGTGGAACGTCATCA-3′/5′-TGTTCCCACAAAATTGAGCA-3′ |
| CZ(8/5)175331520 | 5′-GGCAAAGGGAAAATTGGTG-3′/5′-AGCCTTTATCCCTTTAAGCCA-3′ |
| CZ(9/5)194942758 | 5′-TTCAGCGCTGTTCGTGTC-3′/5′-TCCCGTGTACATGGAGCAT-3′ |
| CZ(10/5)218600291 | 5′-CCAGCGGCCTAAGCATTT-3′/5′-TTGGATCGATCGACATACCT-3′ |
| CZ(11/5)223180071 | 5′-GGCCTCATCAACTCTTGGA-3′/5′-TGACCCACTGCTGCACAT-3′ |
| CZ(0/6)9909329 | 5′-TGCTGCCTTCACCTCGAT-3′/5′-CGAAAATACGGCCCCTGT-3′ |
| CZ(1/6)38164584 | 5′-CAGGTGTACGTAGACGCAGC-3′/5′-CGGCACAAGCAGCTAAAC-3′ |
| CZ(2/6)40024641 | 5′-AACGCTCAGAGTTAGGTGGC-3′/5′-AAACATCAGGGTAAAGGTTCG-3′ |
| CZ(3/6)60273825 | 5′-TTGTGCATCACGGTACAACG-3′/5′-TGCTTACCGCAACATAGTGTC-3′ |
| CZ(4/6)83579108 | 5′-GTGCACCATCGAATGGGA-3′/5′-TAGGACTCATCCTCTGCGG-3′ |
| CZ(5/6)109975180 | 5′-TAGTAGGCACCCCATGCC-3′/5′-TGGGCTCTGAAGGGTTTG-3′ |
| CZ(6/6)136569739 | 5′-TGGCCCATTCATCCATTT-3′/5′-ACGTGTTTGAACCGTGGG-3′ |
| CZ(7/6)159789671 | 5′-TTTGGGCAAAAACAACAGC-3′/5′-CGAGCGTTTTCCATACCG-3′ |
| CZ(8/6)165375001 | 5′-ATGCCCTCGCACATATCG-3′/5′-GTGAATGTCACGCGGGTT-3′ |
| CZ(0/7)14105029 | 5′-TCAAGTGATAGCCGACTTGC-3′/5′-AGCAGCACCACTGGTTCAC-3′ |
| CZ(1/7)22577899 | 5′-TCCATGGGGCGAGTTCTA-3′/5′-GGTGGGGATGATCCATGA-3′ |
| CZ(2/7)49113016 | 5′-TCTTGCGGGTAAGCATCTC-3′/5′-TCATGGGACAGCATGAGC-3′ |
| CZ(4/7)94447252 | 5′-TGCAATACTCCGTGCTTTCA-3′/5′-CCGCACAAATTAAGGGTTG-3′ |
| CZ(5/7)108644147 | 5′-TGAGCAAAACGAATACGCA-3′/5′-AGCGGCCAATGTTGTTTC-3′ |
| CZ(6/7)122735292 | 5′-CTCCCAACAATGATGCCA-3′/5′-TTCAGCGTCCAACATCCA-3′ |
| CZ(7/7)141951558 | 5′-TAACACGCACAGGCAAGC-3′/5′-ATCGCATACAGGGGCAAG-3′ |
| CZ(8/7)161336549 | 5′-GTAATCTGTTTCGCCCCG-3′/5′-AGCATTCTGGTCCCGTGT-3′ |
| CZ(9/7)181059684 | 5′-CTGCATGCCATATTGGGG-3′/5′-TTCACGTGCAGCCATCAC-3′ |
| CZ(0/8)13640513 | 5′-GGCTCGTTCACATGGCTT-3′/5′-CCACGAGCCGCTGATAAT-3′ |
| CZ(1/8)34763204 | 5′-TCTCCAGTTTTTGGACTCCC-3′/5′-ACGTTTCCTTCTTGCCCC-3′ |
| CZ(3/8)76424313 | 5′-TTCGCTCCTGAGACACCC-3′/5′-TTACCGCCCTGTTAGGGA-3′ |
| CZ(4/8)96889577 | 5′-AATCTGCAACCTGCCCCT-3′/5′-AACTCTTGAGGTCCCACGG-3′ |
| CZ(5/8)117486239 | 5′-AGCGCTGCCAATATGAGG-3′/5′-ACACACTCTGACGCTAACGAT-3′ |
| CZ(6/8)123643166 | 5′-CAAGCTGACGGATCACGA-3′/5′-GGGCAGATCACCATTCCTT-3′ |
| CZ(7/8)156375150 | 5′-TGCATCTTTGCCCATCAAT-3′/5′-TCTTTGTGCCCCCGATTA-3′ |
| CZ(8/8)164431517 | 5′-AGGTGCAAATCCGTCGTC-3′/5′-CCGGCCATGATCTGAATC-3′ |
| CZ(9/8)180340009 | 5′-TCGGCACAAATGTAATGACTG-3′/5′-ACCAGGCAGCGCTTGTTT-3′ |
| CZ(0/9)18165088 | 5′-CCTTCGGTTCCCCAATCT-3′/5′-ATTCCGCACAAGCAAAGG-3′ |
| CZ(1/9)25060450 | 5′-ACTGGCGTCAAGTGGTGA-3′/5′-GGAGGGGCACATTGATGA-3′ |
| CZ(2/9)42606416 | 5′-TTGGAGACATCGACAAGGC-3′/5′-GCACCCACTAGAAATGTTCG-3′ |
| CZ(3/9)67893647 | 5′-CACCATTGCATAAGTGTCACC-3′/5′-TCCCACAACAAAAATTCAGC-3′ |
| CZ(4/9)89736083 | 5′-CATTGGCCGATGCTAACC-3′/5′-TTTTACGCCCCGTAATGC-3′ |

| | |
|---|---|
| CZ(5/9)107879235 | 5′-TTAGGCCTTCCCATTCCAG-3′/5′-ACCTCCTCCAAACCTCGG-3′ |
| CZ(6/9)139511877 | 5′-TCCGTCGTGTGTGCAGTT-3′/5′-CATGGCCCAGTGAGAGAGA-3′ |
| CZ(7/9)152296211 | 5′-TGCTTAGCAACACCATGCC-3′/5′-ATGACATAGGTGGTTGCAGTT-3′ |
| CZ(0/10)16004644 | 5′-CCAAAATTTCTTCAGCGCC-3′/5′-TTGGTGGCTGAAGAGGTAACA-3′ |
| CZ(1/10)22391759 | 5′-GCCGCTGAACCATCTTTT-3′/5′-CCCGGCCGCCTATAAAT-3′ |
| CZ(2/10)59782800 | 5′-GCATCGATTGGTCAAGGG-3′/5′-TCCTTGATCGGGTTGTCCT-3′ |
| CZ(3/10)61731546 | 5′-CCCATCGAACCACTCCAG-3′/5′-ATGGCCCAAACCATCCTT-3′ |
| CZ(4/10)87301666 | 5′-TCTAGCCATGGGGGCATA-3′/5′-CGCGACGGATAGATGCTAA-3′ |
| CZ(5/10)117960639 | 5′-CCGATGGTCGTAGATCTGG-3′/5′-TGGCATACCTACAGCGCA-3′ |
| CZ(6/10)133750391 | 5′-AGTGCTCGCCGATAATGG-3′/5′-GGAAAACCCCGCGACTAT-3′ |
| CZ(7/10)145678252 | 5′-CACCAACTCTACGAGCAAAGA-3′/5′-GTTATGCGCTCCACTCCA-3′ |

Note: CZ(0/1)12699720 represents InDel marker at 12699720 bp in bin 0 of chromosome 1 between Chang 7-2 and Zh-1, and so on.

**Table S2** Background recovery rate of 103 $BC_1$ plants between Chang 7-2 and L10

| Plant | Recovery rate | Plant | Recovery rate | Plant | Recovery rate |
|---|---|---|---|---|---|
| $BC_1$-1 | 86.11% | $BC_1$-36 | 81.48% | $BC_1$-71 | 69.44% |
| $BC_1$-2 | 78.70% | $BC_1$-37 | 74.07% | $BC_1$-72 | 77.78% |
| $BC_1$-3 | 73.15% | $BC_1$-38 | 67.59% | $BC_1$-73 | 75.93% |
| $BC_1$-4 | 73.15% | $BC_1$-39 | 75.93% | $BC_1$-74 | 72.22% |
| $BC_1$-5 | 87.04% | $BC_1$-40 | 69.44% | $BC_1$-75 | 74.07% |
| $BC_1$-6 | 81.48% | $BC_1$-41 | 86.11% | $BC_1$-76 | 74.07% |
| $BC_1$-7 | 78.70% | $BC_1$-42 | 75.93% | $BC_1$-77 | 73.15% |
| $BC_1$-8 | 83.33% | $BC_1$-43 | 77.78% | $BC_1$-78 | 74.07% |
| $BC_1$-9 | 68.52% | $BC_1$-44 | 69.44% | $BC_1$-79 | 73.15% |
| $BC_1$-10 | 68.52% | $BC_1$-45 | 83.33% | $BC_1$-80 | 73.15% |
| $BC_1$-11 | 70.37% | $BC_1$-46 | 87.96% | $BC_1$-81 | 77.78% |
| $BC_1$-12 | 67.59% | $BC_1$-47 | 85.19% | $BC_1$-82 | 72.22% |
| $BC_1$-13 | 77.78% | $BC_1$-48 | 69.44% | $BC_1$-83 | 81.48% |
| $BC_1$-14 | 73.15% | $BC_1$-49 | 73.15% | $BC_1$-84 | 69.44% |
| $BC_1$-15 | 75.93% | $BC_1$-50 | 73.15% | $BC_1$-85 | 74.07% |
| $BC_1$-16 | 73.15% | $BC_1$-51 | 71.30% | $BC_1$-86 | 71.30% |
| $BC_1$-17 | 71.30% | $BC_1$-52 | 75.93% | $BC_1$-87 | 75.93% |
| $BC_1$-18 | 71.30% | $BC_1$-53 | 81.48% | $BC_1$-88 | 72.22% |
| $BC_1$-19 | 76.85% | $BC_1$-54 | 80.56% | $BC_1$-89 | 72.22% |
| $BC_1$-20 | 81.48% | $BC_1$-55 | 77.78% | $BC_1$-90 | 74.07% |
| $BC_1$-21 | 75.93% | $BC_1$-56 | 72.22% | $BC_1$-91 | 71.30% |
| $BC_1$-22 | 77.78% | $BC_1$-57 | 73.15% | $BC_1$-92 | 71.30% |
| $BC_1$-23 | 68.52% | $BC_1$-58 | 73.15% | $BC_1$-93 | 66.67% |
| $BC_1$-24 | 68.52% | $BC_1$-59 | 67.59% | $BC_1$-94 | 77.78% |
| $BC_1$-25 | 76.85% | $BC_1$-60 | 68.52% | $BC_1$-95 | 85.19% |
| $BC_1$-26 | 71.30% | $BC_1$-61 | 78.70% | $BC_1$-96 | 69.44% |

| | | | | | |
|---|---|---|---|---|---|
| $BC_1$-27 | 87.96% | $BC_1$-62 | 68.52% | $BC_1$-97 | 76.85% |
| $BC_1$-28 | 75.93% | $BC_1$-63 | 68.52% | $BC_1$-98 | 74.07% |
| $BC_1$-29 | 71.30% | $BC_1$-64 | 68.52% | $BC_1$-99 | 67.59% |
| $BC_1$-30 | 71.30% | $BC_1$-65 | 76.85% | $BC_1$-100 | 68.52% |
| $BC_1$-31 | 73.15% | $BC_1$-66 | 76.85% | $BC_1$-101 | 84.26% |
| $BC_1$-32 | 68.52% | $BC_1$-67 | 75.93% | $BC_1$-102 | 74.07% |
| $BC_1$-33 | 72.22% | $BC_1$-68 | 75.93% | $BC_1$-103 | 69.44% |
| $BC_1$-34 | 85.19% | $BC_1$-69 | 69.44% | | |
| $BC_1$-35 | 80.56% | $BC_1$-70 | 76.85% | | |
